# Naturally acquired antibody response to *Plasmodium falciparum* and *Plasmodium vivax* among indigenous Orang Asli communities in Peninsular Malaysia

**DOI:** 10.3389/fcimb.2023.1165634

**Published:** 2023-04-20

**Authors:** Mohd Amirul Fitri A. Rahim, Mohd Bakhtiar Munajat, Nor Diyana Dian, Mohd Ikhwan Mukmin Seri Rakna, Wathiqah Wahid, Nuraffini Ghazali, Noor Wanie Hassan, Siti Nor Azreen Abdul Manap, Muhd Rafiq Mohd Kasri, Ahmad Imran Mohamed, Emelia Osman, Sriwipa Chuangchaiya, Inke Nadia D. Lubis, Paul C. S. Divis, Akira Kaneko, Kevin K. A. Tetteh, Zulkarnain Md Idris

**Affiliations:** ^1^ Department of Parasitology and Medical Entomology, Faculty of Medicine, Universiti Kebangsaan Malaysia, Cheras, Kuala Lumpur, Malaysia; ^2^ District Health Office of Gua Musang, Gua Musang, Kelantan, Malaysia; ^3^ Department of Community Health, Faculty of Public Health, Kasetsart University, Sakon Nakhon, Thailand; ^4^ Department of Paediatric, Faculty of Medicine, Universitas Sumatera Utara, Medan, Indonesia; ^5^ Malaria Research Centre, Faculty of Medicine and Health Sciences, Universiti Malaysia Sarawak, Kota Samarahan, Sarawak, Malaysia; ^6^ Department of Microbiology, Tumor and Cell Biology, Karolinska Institutet, Stockholm, Sweden; ^7^ Department of Parasitology, Graduate School of Medicine, Osaka City University, Osaka, Japan; ^8^ Department of Infection Biology, Faculty of Infectious Tropical Diseases, London School of Hygiene and Tropical Medicine, London, United Kingdom

**Keywords:** malaria, serology, *Plasmodium falciparum*, *Plasmodium vivax*, Orang Asli, Malaysia, indigenous

## Abstract

Malaria remains a public health problem in many parts of the world. In Malaysia, the significant progress towards the national elimination programme and effective disease notification on malaria has resulted in zero indigenous human malaria cases since 2018. However, the country still needs to determine the extent of malaria exposure and transmission patterns, particularly in high-risk populations. In this study, a serological method was used to measure transmission levels of *Plasmodium falciparum* and *Plasmodium vivax* among indigenous Orang Asli communities in Kelantan, Peninsular Malaysia. A community-based cross-sectional survey was conducted in three Orang Asli communities (i.e., Pos Bihai, Pos Gob, and Pos Kuala Betis) in Kelantan from June to July 2019. Antibody responses to malaria were assessed by enzyme-linked immunosorbent assay (ELISA) using two *P. falciparum* (PfAMA-1 and PfMSP-1_19_) and two *P. vivax* (PvAMA-1 and PvMSP-1_19_) antigens. Age-adjusted antibody responses were analysed using a reversible catalytic model to calculate seroconversion rates (SCRs). Multiple logistic regression was used to investigate factors associated with malaria exposure. The overall malaria seroprevalence was 38.8% for PfAMA-1, 36.4% for PfMSP-1_19_, 2.2% for PvAMA-1, and 9.3% for PvMSP-1_19_. Between study areas, the proportion of seropositivity for any *P. falciparum* and *P. vivax* antigens was significantly highest in Pos Kuala Betis with 34.7% (*p* < 0.001) and 13.6% (*p* < 0.001), respectively. For all parasite antigens except for PvAMA-1, the proportion of seropositive individuals significantly increased with age (all *p* < 0.001). Based on the SCR, there was a higher level of *P. falciparum* transmission than *P. vivax* in the study area. Multivariate regression analyses showed that living in Pos Kuala Betis was associated with both *P. falciparum* (adjusted odds ratio [aOR] 5.6, *p* < 0.001) and *P. vivax* (aOR 2.1, *p* < 0.001) seropositivities. Significant associations were also found between age and seropositivity to *P. falciparum* and *P. vivax* antigens. Analysis of community-based serological data helps describe the level of transmission, heterogeneity, and factors associated with malaria exposure among indigenous communities in Peninsular Malaysia. This approach could be an important adjunct tool for malaria monitoring and surveillance in low malaria transmission settings in the country.

## Introduction

Malaysia achieved a remarkable reduction in malaria morbidity and mortality following many decades of control efforts. In 2020, no indigenous human malaria cases were reported in Malaysia for the third consecutive year (WHO, 2020). Although numbers of zoonotic malaria due to *Plasmodium knowlesi* continue to be reported in the country (Rahim et al., 2020; Dian et al., 2022a; Muhammad et al., 2022), consistent efforts to maintain zero indigenous cases, mainly from *Plasmodium falciparum* and *Plasmodium vivax*, and achieve the aim of malaria-free status are still the main priority of the government. As malaria transmission declines, efforts to find the sparse and heterogeneously distributed remaining infections in Malaysia could result in operational challenges. Therefore, in order to consolidate gains and progress to elimination, improved tools for infection monitoring are required.

Conventional surveillance methods such as parasite prevalence and entomological inoculation rates (EIRs), of which the EIR is considered the gold standard for assessing malaria transmission intensity, are no longer applicable in areas with low-intensity malaria transmission due to their low sensitivity (Drakeley et al., 2005; Achee et al., 2015). Unlike many other infectious diseases, malaria antibodies against parasite antigens are widely divergent; some may last for a longer time than others (Guerin et al., 2002; Loker et al., 2004). Antibody status may not reflect the active cases, but serology has been recommended as a sensitive and reliable tool for evaluating the level of immunity and the intensity of malaria transmission in populations (Rosado et al., 2021). Uniquely, serology also allows retrospective examination of exposure history, including the effects of interventions and the absence of recent exposure in elimination settings (Drakeley et al., 2005; Stewart et al., 2009). Furthermore, seropositivity to parasite antigens in a particular community is believed to represent cumulative exposure to prior malaria cases, decreasing the likelihood of missing exposure events, even silent infections (Baum et al., 2016). Additionally, by examining age-seroprevalence patterns, it is feasible to pinpoint historical patterns in the level of transmission in a particular population group and how these patterns vary throughout groups (Rahim et al., 2022). This approach also has been utilised in many other countries and reported as a more sensitive tool to evaluate population-level malaria exposure in low malaria transmission or areas in the pre-elimination phase due to its high sensitivity (Idris et al., 2017a; Keffale et al., 2019; Surendra et al., 2019; Rahim et al., 2022).

As countries go through the epidemiological transition from high through moderate to low transmission, it becomes challenging to assess intervention programme performance and to know when it is appropriate to conduct an impact evaluation. Therefore, the seroepidemiological analysis would benefit malaria control programmes if implemented and evaluated in regions on the path to elimination as Malaysia. This study was designed to determine serological transmission levels of *P. falciparum* and *P. vivax* among the most vulnerable population to malaria infection in Malaysia, namely, the indigenous Orang Asli. Results from this study could be an important additional tool for malaria monitoring and surveillance in high-risk and low-transmission settings in Malaysia and other countries.

## Materials and methods

### Ethics statement

The study was conducted in accordance with the Declaration of Helsinki and was approved by the Medical Ethics Committee of the National University of Malaysia (reference number UKM PPI/111/8/JEP-2019-148). Permission for fieldwork was obtained from the Regional Health Office of Kelantan (reference number JKNK/BPA/KV 01/2/1). Respondents were sensitised to the study objectives and procedures by the local health district personnel for the study participation.

### Study area and sample collection

**Figure 1:**
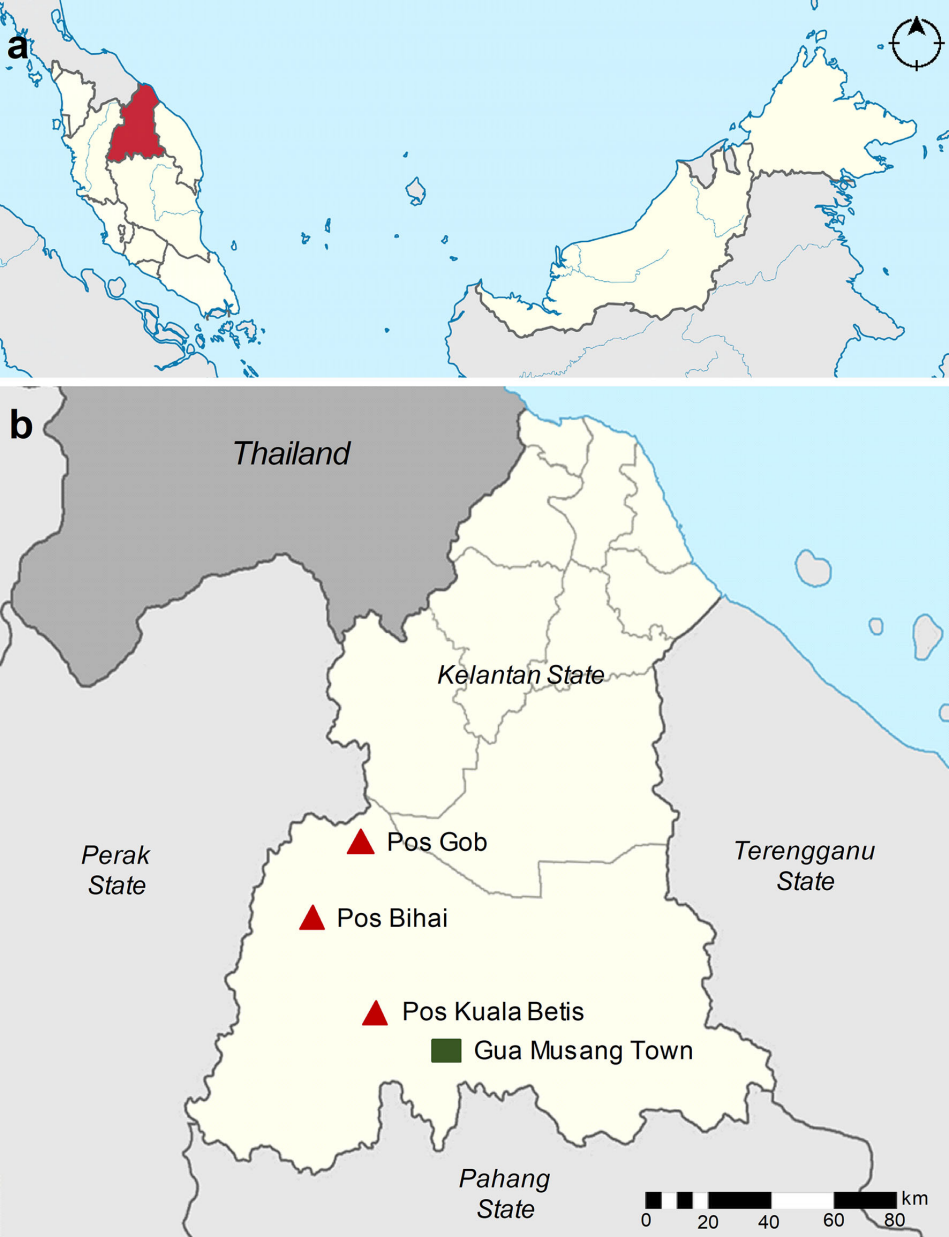
Map of the study area. **(A)** Map of Malaysia showing the location of Kelantan state (red). **(B)** The location of the three study villages is within the Gua Musang district, Kelantan state (beige). The study villages, namely, Pos Kuala Betis, Pos Bihai, and Pos Gob, are indicated in the red triangle, and the nearby town Gua Musang is indicated in the green square.

**Figure 2:**
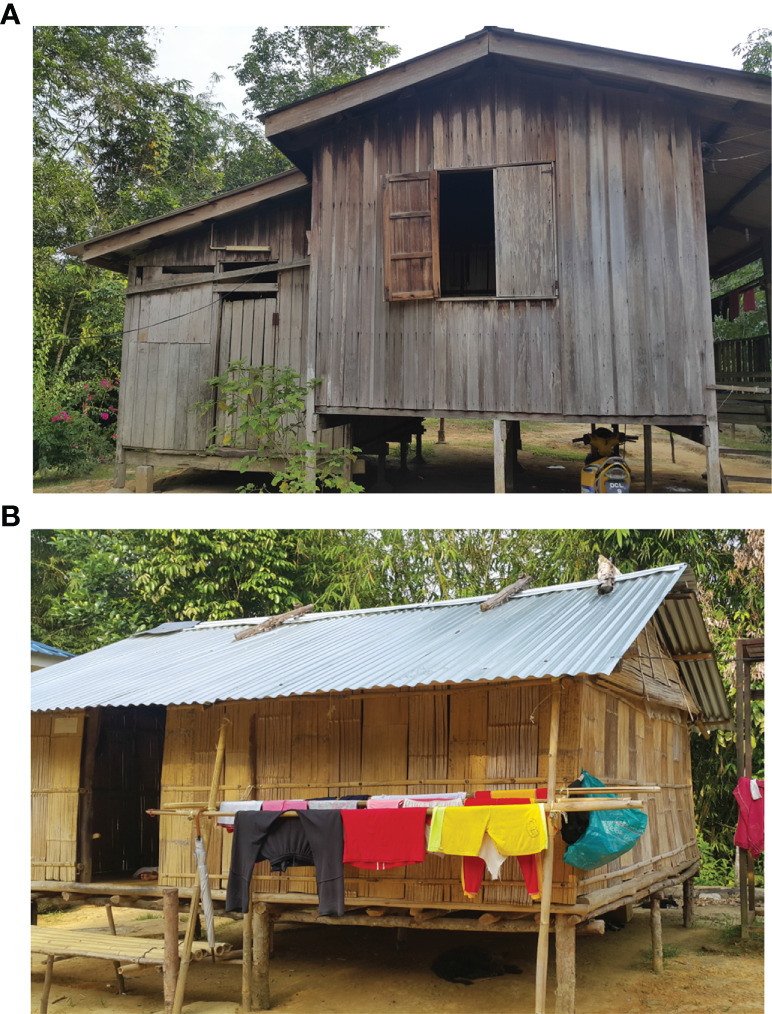
Example of different housing styles in the study area. **(A)** A house constructed of wood wall and floor by the government and **(B)** an elevated makeshift house constructed of bamboo wall and floor, with a corrugated iron roof. Photographs were taken by author Zulkarnain Md Idris.

The study was conducted in three indigenous Orang Asli settlements, namely, Pos Kuala Betis (4.5322°N; 101.4530°E), Pos Bihai (5.03623°N; 101.63155°E), and Pos Gob (5.28080°N; 101.66641°E) located in Gua Musang district, Kelantan state, Peninsular Malaysia (**Figure 1**). In these settlements, all participants belong to the Temiar sub-ethnic group of Senoi. Generally, the topography of the area is a hilly terrain elevated at 500–1,000 above sea level. The study area has a typical tropical monsoon climate where it receives abundant rainfall amount that exceeds 2,500 mm/year and a mean annual temperature of approximately 27.5°C (Tew et al., 2022). The surrounding area is dominantly forest, followed by partial cropland for rubber, oil palm, and vegetable plantations. The communities are traditionally and culturally forest-dependent people who move in a semi-nomadic manner acquiring various resources in the tropical forests for subsistence and livelihood in a sustainable manner (Munajat et al., 2021). In terms of housing style, most houses were typically elevated houses provided free by the government and constructed of wooden materials (i.e., wall and floor) with corrugated iron roofs. However, some residents still prefer living in makeshift bamboo houses (Munajat et al., 2021). An example of the different housing styles in the study settlements is shown in **Figure 2**.

A cross-sectional survey was carried out between June and July 2019. All villagers were invited to take part in the study. The study protocol and consent were explained to the participants, and their voluntary consent was documented. For the illiterate participant, written informed consent was obtained in the presence of an independent literate witness. Written informed consent was obtained from parents or legal guardians for children (i.e., between 6 months and 9 years old) and adolescents (between 10 and 18 years old). Finger-prick blood samples were obtained from each participant for microscopy smears and filter-paper blood spots, and the latter were stored at −20°C until processing. District healthcare providers and community leaders (i.e., Tok Batin) were purposely involved in the study to facilitate participation and cooperation among the community.

### Microscopy examination

Giemsa-stained thick and thin malaria films reading was performed by trained laboratory technicians to identify active infections. All blood films were stained with 3% Giemsa solution (Merck, Darmstadt, Germany) for 30 min and examined under oil emersion (10 × 100 magnification). Blood smears were negative if no parasites were found after examining 100 high-power microscopy fields.

### Assessment of anti-malaria antibody responses

Blood spots were reconstituted as previously described, and the solution was equivalent to a 1:200 dilution of serum (Idris et al., 2017a; Idris et al., 2017b; Rahim et al., 2022). Antibody responses against apical membrane antigen-1 or the 19-kDa fragment of merozoite surface protein-1 for *P. falciparum* (PfAMA-1 and PfMSP-1_19_, respectively) and *P. vivax* (PvAMA-1 and PvMSP-1_19_) were determined by ELISA as previously described (Idris et al., 2017a; Idris et al., 2017b; Rahim et al., 2022). Briefly, sera from the reconstituted blood spot samples were added in duplicate at a final concentration of 1:1,000 for MSP-1_19_ and 1:2,000 for AMA-1. In addition, four blank wells and a fivefold dilution series (starting at 1:100 for AMA-1 and 1:50 for MSP-1_19_) of a hyper-immune plasma pool (n = 15) were added per plate. Optical density (OD) values were measured at 450 nm with a Multiskan Go ELISA microplate reader (Thermo Scientific, USA).

### Statistical analysis

The gathered data were compiled into a Microsoft Excel spreadsheet and cross-checked for errors. All further statistical analyses were done in STATA version 13.1 (StataCorp, TX, USA). The median and interquartile range (IQR) were used to provide data for continuous variables, while frequencies and percentages were used to present data for categorical variables. Differences in proportions were tested using the chi-squared test. Antibody levels between categories were compared using the Mann–Whitney U test or the Kruskal–Wallis test with Dunn’s multiple comparison *post-hoc* tests. Duplicate ODs per individual were averaged, adjusted for background reactivity, and normalised against the positive control curve as previously described to adjust for plate variation (Corran et al., 2008). Thresholds to define seropositivity for the separate antigens were calculated using a finite mixture model (Stewart et al., 2009). Individuals were defined as seropositive when their adjusted OD value would be greater than the mean of the lower Gaussian distribution plus three times the standard deviation. The reversible catalytic model was used to define the seroconversion rate (SCR) and to plot corresponding seroconversion curves while fitting age-adjusted seropositivity to *P. falciparum* or *P. vivax* using maximum likelihood (Drakeley et al., 2005). Infants under 1 year of age were excluded from the reversible catalytic model to remove any influence of maternally derived antibodies (Drakeley et al., 2005). Factors associated with *P. falciparum* and *P. vivax* seropositivities were determined independently for each site using generalised estimating equations adjusting for correlation between observations from the same variables. Variables that were significant at *p* < 0.10 in the univariate analyses were incorporated into the multivariate model and retained in the final multivariate model if their association with immune responses was statistically significant at *p* < 0.05.

## Results

### General characteristics of the study population and parasite detection

Serum was successfully eluted and serologically tested for 645 participants in this study (**Table 1**). The majority of participants were women (54.4%), aged 16 and 30 years (30.1%) with a median age of 21 years old and from Pos Kuala Betis (49.9%). Examination of microscopy slides found no malaria infections.

**Table 1:** General characteristics of the study population of indigenous Orang Asli communities in Gua Musang district, Kelantan state, Peninsular Malaysia.

Characteristics	n (%)
Total number of participants	645 (100)
Gender	
Male	294 (45.6)
Female	351 (54.4)
Age, median (IQR), years	21 (7-35)
Age group, years	
≤5	122 (18.9)
6–15	138 (21.4)
16–30	194 (30.1)
>30	191 (29.6)
Study settlement	
Pos Kuala Betis	322 (49.9)
Pos Bihai	164 (25.4)
Pos Gob	159 (24.7)

IQR = interquartile range.

### Breadth of antibody responses

**Figure 3:**
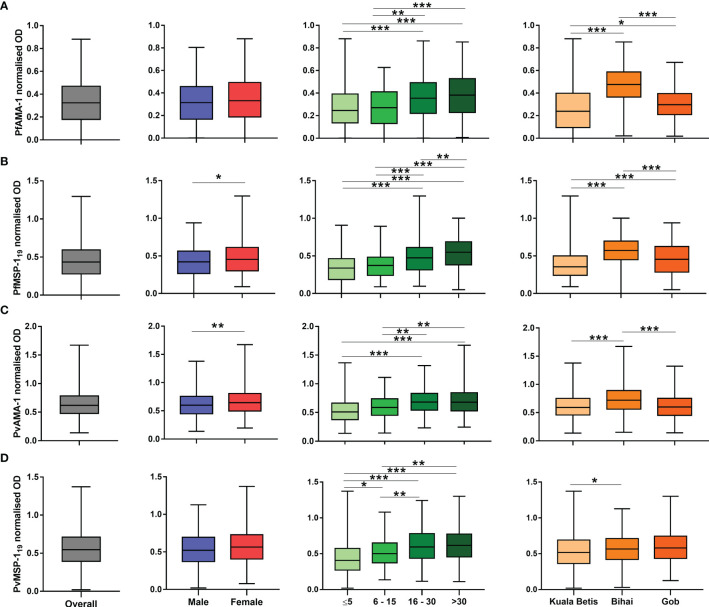
Distribution of antibody responses to *P. falciparum* and *P. vivax* antigens expressed as normalised optical density (OD) values. Plots are divided by antigen: **(A)** PfAMA-1, **(B)** PfMSP-1_19_, **(C)** PvAMA-1, and **(D)** PvMSP-1_19_. The interquartile range (IQR, 25–75th percentile) is represented in a box plot with the median OD value shown as a line within the box. Antibody levels between categories were compared using the Mann–Whitney U test or the Kruskal–Wallis test with Dunn’s multiple comparison *post-hoc* tests.*p < 0.05; **p < 0.01; ***p < 0.001.

Antibody levels as measured in optical densities are shown in **Figure 3**. Overall, the median antibody levels showed a significant difference between antigens (*p* < 0.001). Except for PfMSP-1_19_ (*p* = 0.043) and PvAMA-1 (*p* = 0.008), no significant differences between genders were observed in PfAMA-1 and PvMSP-1_19_ median antibody levels. As expected, median antibody levels significantly differed between age groups and increased significantly with age (*p* < 0.001) for all antigens. Furthermore, median antibody levels to all antigens also differed significantly (*p* < 0.05) between settlements. For *P. falciparum*, the anti-AMA-1 and anti-MSP-1_19_ levels were significantly (*p* < 0.05) higher among the population in Pos Bihai and Pos Gob than in Pos Kuala Betis. For *P. vivax*, the anti-AMA-1 antibody level was significantly (*p* < 0.001) higher among the population in Pos Bihai than in the other two settlements. In comparison, only the anti-MSP-1_19_ antibody level was significantly (*p* = 0.042) higher in Pos Bihai than in Pos Kuala Betis.

### Seroprevalence and seroconversion rates

**Table 2** shows the overall seroprevalence of *P. falciparum* and *P. vivax* antigens among indigenous Orang Asli communities in Kelantan. Overall, malaria seroprevalence was 38.8% for PfAMA-1, 36.4% for PfMSP-1_19_, 2.2% for PvAMA-1, and 9.3% for PvMSP-1_19_. Seropositivity to all parasite antigens was generally higher in women than in men, although no significant difference was observed (*p* = 0.378). For all parasite antigens, the proportion of seropositive individuals significantly increased with age (*p* = 0.004), highest in the >30 age group and lowest in the ≤5 age group. Between study areas, the proportion of seropositive individuals was significantly different (*p* < 0.001) for all parasite antigens. The highest proportion of seropositive individuals was observed in Pos Kuala Betis for PfAMA-1, PvMSP-1_19_, and PvAMA-1 antigens, with 26.5%, 8.8%, and 1.2%, respectively. In Pos Bihai, 18.7% and none were seropositive to PfMSP-1_19_ and PvMSP-1_19_ antigens, respectively.

**Table 2:** Anti-malarial seropositivity for participants in three indigenous communities (N=645) in Gua Musang district, Kelantan state, Peninsular Malaysia.

Characteristics	PfAMA-1	PfMSP-1_19_	PvAMA-1	PvMSP-1_19_	*p*-value
Overall, n (%)	250 (38.8)	235 (36.4)	14 (2.2)	60 (9.3)	
Gender, n (%)					
Male	100 (15.5)	97 (15.1)	4 (0.6)	24 (3.7)	0.378
Female	150 (23.3)	138 (21.3)	10 (1.6)	36 (5.6)	
Age group, n (%)					
≤5	14 (2.2)	11 (1.7)	0 (0.0)	2 (0.3)	0.004
6–15	45 (6.9)	21 (3.3)	0 (0.0)	18 (2.8)	
16–30	94 (14.6)	95 (14.7)	6 (0.9)	17 (2.6)	
>30	97 (15.1)	108 (16.7)	8 (1.3)	23 (3.6)	
Study settlement, n (%)					
Pos Kuala Betis	171 (26.5)	31 (4.8)	8 (1.2)	57 (8.8)	<0.001
Pos Bihai	75 (11.7)	121 (18.7)	1 (0.2)	0 (0.0)	
Pos Gob	4 (0.6)	83 (12.9)	5 (0.8)	3 (0.5)	

Differences in proportions were tested using the chi-squared test.

n = number of seropositive.

**Figure 4:**
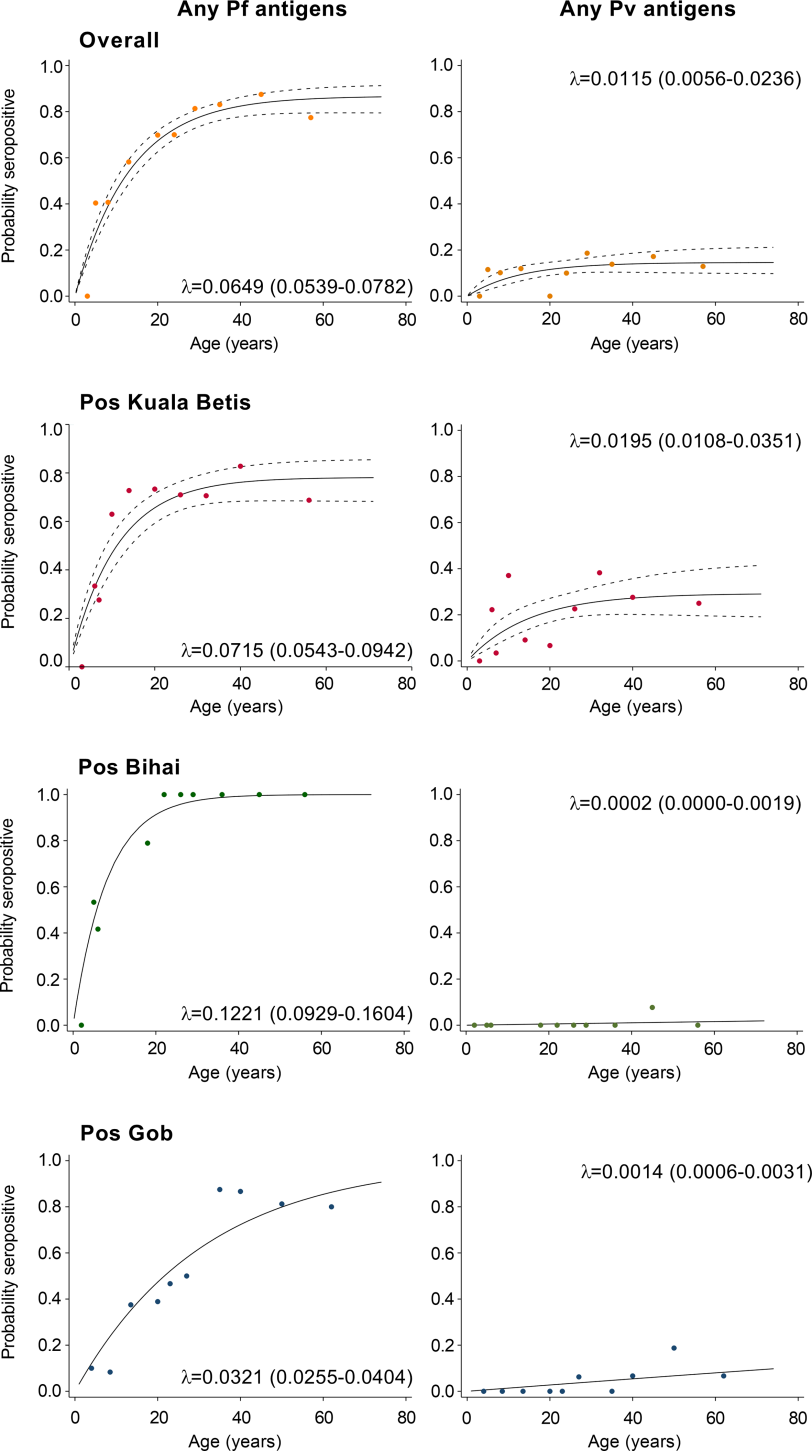
Annual probability of malaria seroconversion rate in Gua Musang district, Kelantan state. *P. falciparum*- and *P. vivax*-specific antigens by age in each setting. Points indicated observed seroprevalence, and solid lines show model-predicted seroprevalence. The broken line represents 95% confidence intervals. Seroconversion rates (SCRs; λ) are presented on the graph.

To define any serological evidence of exposure to *P. falciparum* or *P. vivax*, seropositivity to species-specific AMA-1 and MSP-1_19_ antigens was combined. In total, 58.3% of participants were seropositive for *P. falciparum* (375/645), 10.1% for *P. vivax* (65/645), and 7.9% for both species (51/645). Relatedly, the annual probability of malaria seroconversion curves based on the combination of seropositivity to species-specific AMA-1 and MSP-1_19_ antigens are shown in **Figure 4**. Based on the SCRs, there was a higher level of *P. falciparum* transmission than *P. vivax* using all tested antigens, ranging from 0.032 year^−1^ (0.026–0.04) to 0.122 year^−1^ (0.093–0.16) for any *P. falciparum* and 0.000 year^−1^ (0.000–0.002) to 0.019 year^−1^ (0.011–0.035) for any *P. vivax*. The SCRs were statistically significant between species-specific antigens, as evidenced by the non-overlapping confidence intervals.

### Factors associated with seropositivity

Univariate and multivariate logistic regression analyses to identify factors associated with seropositivity to any *P. falciparum-* and *P. vivax*-specific antigens are shown in **Table 3**. The likelihood of seropositivity increased significantly with age for both *P. falciparum* and *P. vivax* antigens (all *p* < 0.05). Significant associations were also found between settlement and seropositivity in the adjusted model for *P. falciparum* and *P. vivax* antigens. For *P. falciparum*, a significantly higher risk of seropositive was observed in Pos Bihai (adjusted odds ratio [aOR] 2.75, 1.67–4.51, *p* < 0.001) but a lower risk of seropositive in Pos Gob (aOR 0.49, 0.31–0.79, *p* = 0.003), when compared to Pos Kuala Betis. In contrast, a significantly lower risk of *P. vivax* seropositivity remained apparent in the adjusted model for Pos Bihai (aOR 0.02, 0.00–0.18, *p* < 0.001) and Pos Gob (aOR 0.14, 0.06–0.34, *p* < 0.001), when compared to Pos Kuala Betis. No association was found between gender to seropositivity for *P. falciparum* and *P. vivax* antigens.

**Table 3:** Logistic regression analysis of seropositivity to any *P. falciparum-* and *P. vivax*-specific antigens among indigenous Orang Asli communities in Kelantan state, Peninsular Malaysia.

Variable	Category	n/N	*P. falciparum* (PfAMA-1 + PfMSP-1_19_)				n/N	*P. vivax* (PvAMA-1 + PvMSP-1_19_)			
			Crude OR (95% CI)	*p*-value	Adjusted OR (95% CI)	*p*-value		Crude OR (95% CI)	*p*-value	Adjusted OR (95% CI)	*p*-value
Gender	Male	160/294	1.00		1.00		26/294	1.00		1.00	
	Female	216/351	1.34 (0.98-1.84)	0.068	1.26 (0.87-1.82)	0.219	39/351	1.29 (0.76-2.17)	0.342	1.26 (0.73-2.17)	0.409
Age group	≤5	19/122	1.00		1.00		2/122	1.00		1.00	
	6-15	57/138	3.81 (2.11-6.92)	<0.001	3.66 (2.01-6.65)	<0.001	18/138	8.99 (2.04-39.64)	0.004	7.45 (1.68-33.16)	0.008
	16-30	142/194	14.81 (8.26-26.53)	<0.001	15.12 (8.37-27.31)	<0.001	17/194	5.76 (1.31-25.41)	0.021	6.82 (1.52-30.51)	0.012
	>30	158/191	25.96 (14.01-48.08)	<0.001	27.67 (14.81-51.71)	<0.001	28/191	10.31 (2.41-44.11)	0.002	13.43 (3.09-58.24)	0.001
Setting	Pos Kuala Betis	172/322	1.00		1.00		58/322	1.00		1.00	
	Pos Bihai	121/164	2.45 (1.63-3.71)	<0.001	2.75 (1.67-4.51)	<0.001	1/164	0.03 (0.00-0.21)	<0.001	0.02 (0.00-0.18)	<0.001
	Pos Gob	83/159	0.95 (0.65-1.39)	0.802	0.49 (0.31-0.79)	0.003	6/159	0.18 (0.08-0.42)	<0.001	0.14 (0.06-0.34)	<0.001

Odd ratios (ORs) and their 95% confidence intervals (95% CIs) are presented for both univariate (crude) and multivariate (adjusted) models. Statistical significance was determined using the likelihood ratio test. N = number of individuals in each category; n = number of seropositive for any *P. falciparum* or *P. vivax* specific antigens.

## Discussion

Since the implementation of the Malaria Eradication Program in Malaysian Borneo in 1961 and Peninsular Malaysia in 1967, there has been a significant decrease in malaria cases in Malaysia (Dian et al., 2022a). In 1966, the number of malaria cases in the country was estimated at approximately 300,000. However, by 1977, this number had significantly reduced to an estimated 44,910 cases across the country (WHO, 2015). The decline is attributed to enhanced coverage of indoor residual spraying (IRS) and better passive case detection and management practices (WHO, 2015). Subsequently, the program was strengthened, and malaria has been on the national list of notifiable diseases since 1988 (Dian et al., 2021). Despite having zero indigenous human malaria cases in Malaysia since 2018 (WHO, 2020; Dian et al., 2022b), malaria is still one of the public health problems. Malaria cases in the country predominate in rural populations and reflect social and economic aspects that are accentuated in indigenous populations such as the Orang Asli communities. Between the 80s and 90s, malaria serological studies on *P. falciparum* and *P. vivax* among the Orang Asli communities in four states in Peninsular Malaysia, namely, Kelantan (Thomas et al., 1981), Selangor (Mak et al., 1987), Pahang (Archibald et al., 1990), and Perak (Lee et al., 1988; Gordon et al., 1991), using less sensitive markers such as schizont extract and circumsporozoite protein, reported high seroprevalence of malaria ranging from 76% to 96%. However, after the malaria seroepidemiological study by Gordon et al. in 1991, no serological study of malaria has been conducted among the Orang Asli communities for the past 30 decades. Furthermore, more information is needed on whether the transmission levels of *P. falciparum* and *P. vivax* in the community has changed since 2018. In this study, serological-based transmission levels of *P. falciparum* and *P. vivax* malaria using AMA-1 and MSP-1_19_ were determined among indigenous Orang Asli communities in Kelantan state, Malaysia. The present study indicates significant local variation in malaria seroprevalence between study areas, and the proportion of seropositive individuals increased with age. The results also show that the seropositivity and transmission level based on SCR was higher for *P. falciparum* than for *P. vivax*.

The serological investigation in the present study revealed that humoral antibodies against *P. falciparum* antigens were higher than *P. vivax* antigens (i.e., 58% and 10%, respectively). This finding is consistent with previously described levels in other countries in the Southeast Asia region with *P. falciparum*–*P. vivax* co-endemic, such as Thailand (79% and 40%) (Rahim et al., 2022), Vietnam (38% and 31%) (San et al., 2022), Myanmar (30% and 14%) (Edwards et al., 2021), and Indonesia (6.9% and 2.0%) (Surendra et al., 2019). The higher seropositivity from *P. falciparum* may be due to the fact that serological assay used was originally developed to detect responses to PfAMA-1 and PfMSP-1_19_ antigens and thus may be more sensitive in detecting this species (Tadesse et al., 2017; Björkman et al., 2019; Edwards et al., 2021). Furthermore, *P. falciparum* also circulates at higher parasite densities that induce higher antibody levels than *P. vivax* (Uplekar et al., 2017), and this is likely to produce more detectable antibody levels (Cook et al., 2012; Edwards et al., 2021). Moreover, antibodies against *P. vivax* are acquired quickly but are less durable than antibodies against *P. falciparum*, which accumulate and degrade slowly over time (Yap et al., 2021). Given recent evidence of high levels of antibody cross-reactivity, particularly for MSP-1_19_ between *P. vivax* and *P. knowlesi* (>81% sequence identity between the Sal1 and H strains, respectively) in patients from Malaysia (Longley et al., 2022), further analyses could be conducted to run the serological assay against more antigenic targets to optimise *P. vivax* antibody detection. Hence, it will be important to reduce the potential misclassification of *Plasmodium* species by serology in co-endemic areas.

There was no parasitological or serological evidence of recent local transmission in the present study. This observation indicated both an absence of microscopic infections and weak or absent serological response, particularly for *P. vivax* in children up to the age of 5 years (**Table 2**). These findings reflect the successful impact of the malaria control programme implemented among the indigenous Orang Asli communities in the study area by the health authority. Furthermore, the likelihood of seropositivity increased significantly with age for both *P. falciparum* and *P. vivax* (**Figure 3** and **Table 3**), which clearly indicated age-acquired immunity. It has been known that immune response increases with age and accumulates over time due to long-term exposure to the malaria parasite (Drakeley et al., 2005; Griffin et al., 2015; Stanisic et al., 2015). When immunity to malaria is acquired, antibodies can persist for several years (Julien and Wardemann, 2019). Thus, with every new malaria exposure, the long-lived plasma cells are able to rapidly produce antibodies against *Plasmodium* parasites (Hviid et al., 2015).

The present study is, to our knowledge, the first to use age-adjusted seropositivity data to estimate transmission intensity (i.e., SCR) for *P. falciparum* and *P. vivax* in Malaysia. In this study, the overall *P. falciparum* and *P. vivax* SCRs were 0.065 and 0.012 per year, respectively, implying that only 6.5/100 and 1.2/100 sampled participants previously become seropositive to malaria each year. This result suggests a low force of infection with previously intense transmission and the predominance of *P. falciparum* species exposure in the area. In the area where there was a lack of evidence of infections by microscopy as in the present study, SCR based on serology can offer a more complete picture of the dynamics of malaria transmission. This is because serological tool incorporates exposure over time and reflects cumulative exposure rather than a single current infection (Edwards et al., 2021; Tayipto et al., 2022). Furthermore, because the antibody response to blood-stage antigens is long-lasting and results in higher seroprevalence rates than equivalent parasite rates (Drakeley et al., 2005), SCR has high sensitivity even at low transmission intensities (Cook et al., 2012; Idris et al., 2017a).

As no parasite infections were detected with microscopy, risk factors to describe exposure to malaria among the indigenous Orang Asli could only be examined using serological outcomes. The increased likelihood of seropositivity to *P. falciparum* remained twice as high for Pos Bihai compared with Pos Kuala Betis in the adjusted model. In contrast, the likelihood of seropositivity to *P. vivax* remained low for both Pos Bihai and Pos Gob compared with Pos Kuala Betis in the adjusted model (**Table 3**). Although these settlements belong to the same district, they differ greatly in their location and access to the general population. For instance, Pos Bihai and Pos Gob communities live the furthest in a forested area as compared to Pos Kuala Betis in a forest fringe with easy access to a nearby town (**Figure 1**). It seems that “previous” *P. vivax* transmission in this study was lower in more remote settlements than in the forest fringe area, indicating lower potential receptivity for the re-introduction of malaria in the settlements in this district. The heterogeneity in malaria exposure within communities due to the difference in location has also been reported in other settings (Rosas-Aguirre et al., 2013; Idris et al., 2017b; Kale et al., 2019; Zakeri et al., 2016; Labadie-Bracho et al., 2020; Rahim et al., 2022). Therefore, further monitoring of seroprevalence and population movement between different settlements is considered highly effective, given the different risks of malaria transmission within the study area.

Although this study describes the potential use of serological data analysis in estimating malaria transmission intensity and factors associated with disease exposure among the indigenous communities in Malaysia, the results generated would need to be carefully interpreted. The most obvious one concerns the relatively small number of individuals sampled in each settlement. Although small sample sizes may be sufficient to indicate a considerable reduction in SCR (Sepúlveda et al., 2015a), they invariantly lead to lower estimation precision of the present SCR and prevent the reverse catalytic model from detecting significant changes in malaria transmission over time (Sepúlveda and Drakeley, 2015b). In addition, the study samples were collected using a convenience sampling method. Although this approach is valid in obtaining an estimate of malaria seroprevalence (Stewart et al., 2009), it can overestimate previous malaria incidence among indigenous Orang Asli communities in the study area. Another limitation is the reliance on microscopy as the sole diagnosis method of current infection. Although microscopy examination is considered the gold standard for malaria diagnosis in Malaysia, submicroscopic malaria infections might have been missed in the communities without being accompanied by molecular methods. A further limitation is that the present study is restricted to few sociodemographic variables among participants. Therefore, future studies measuring population-level malaria antibody responses incorporated with additional data collection (e.g., behavioural and household) could describe the more in-depth associated risk of exposure. These additional data could be epidemiologically informative to assist the management of malaria control programs or any potential malaria outbreak in the communities.

## Conclusions

Together, these data suggest that antibody responses to recombinant AMA-1 and MSP-1_19_ antigens for both *P. falcip*arum and *P. vivax* demonstrate the utility in describing variations in endemicity and risk of malaria exposure among indigenous communities in Malaysia and elsewhere. In the absence of active local transmission detected by the conventional method, the serological analysis could be an essential adjunct tool to estimate malaria transmission intensity in areas that lack baseline data. This further demonstrates the potential use of serological metrics in monitoring changes in malaria transmission as countries shift from control to elimination strategies or from indigenous human malaria to zoonotic malaria species.

## Data availability statement

The original contributions presented in the study are included in the article/supplementary material. Further inquiries can be directed to the corresponding author.

## Ethics statement

The studies involving human participants were reviewed and approved by Research and Ethics Committee of Universiti Kebangsaan Malaysia (Reference No. UKM PPI/111/8/JEP-2019-148). Written informed consent to participate in this study was provided by the participants’ legal guardian/next of kin.

## Author contributions

Conceived and designed the study: ZI, KT, PD, IL, SC and MK. Collected and prepared samples: MAR, MM, ND, MSR, NG, NH, SM, MK, AM, EO, WW and ZI. Performed the laboratory assays: MAR, MM, ND, SM and ZI. Analysed the data: MAR, MM, ND and ZI. Contributed reagents, materials, and analysis tools: AK, KT and ZI. Drafted the manuscript: MAR. Provided critical revisions: ZI, KT, AK, PD, IL, SC, EO and WW. All authors contributed to the article and approved the submitted version.
